# Macula- Versus Disc-Centered Fundus Photography: Performance in Age-Prediction and Disease Associations

**DOI:** 10.1167/iovs.67.6.24

**Published:** 2026-06-15

**Authors:** Alexander C. Heatley, Mert Enbiyaoglu, Justin Engelmann, Yukun Zhou, Mario Cortina-Borja, Anthony P. Khawaja, Alastair K. Denniston, Lisa Zhuoting Zhu, Pearse A. Keane, Siegfried K. Wagner

**Affiliations:** 1 Institute of Ophthalmology, University College London, London, United Kingdom; 2 NIHR Biomedical Research Centre, Moorfields Eye Hospital NHS Foundation Trust & UCL Institute of Ophthalmology, London, United Kingdom; 3 Great Ormond Street Institute of Child Health, University College London, London, United Kingdom; 4 Centre for Regulatory Science and Innovation, Birmingham Health Partners, Birmingham, United Kingdom; 5 Center for Eye Research Australia, Royal Victorian Eye and Ear Hospital, East Melbourne, Australia

**Keywords:** retinal age gap (RAG), oculomics, deep learning (DL), fundus photography, macula- and disc-centered imaging

## Abstract

**Purpose:**

The purpose of this study was to compare deep learning (DL)-based age prediction performance and inter-method agreement using macula- versus disc-centered color fundus photographs (CFPs), and to determine whether image fixation modifies the association between retinal age gap (RAG) and disease.

**Methods:**

This retrospective cohort study analyzed 12,230 same-day macula- and disc-centered CFP pairs from 5895 patients (≥40 years) attending Moorfields Eye Hospital (2008–2018). We used a previously validated model that predicts chronological age using either macula- or disc-centered CFPs. DL-predicted age was compared via mean absolute error (MAE), intraclass correlation coefficients (ICCs), and Bland–Altman analysis. Associations between RAG and clinical conditions were assessed using generalized linear mixed-effects models with a patient-level random intercept.

**Results:**

Macula-centered CFPs yielded a smaller MAE than disc-centered images (7.09 vs. 7.77 years), and age predictions differed significantly between fixation types (*P* < 0.01). Overall agreement between macula- and disc-centered predictions was high, with an ICC of 0.83 (95% confidence interval [CI] = 0.82–0.84). RAG was significantly associated with age-related macular degeneration (AMD; odds ratio [OR] = 3.57, 95% CI = 1.76–7.24, *P* < 0.001). No statistically significant interaction between RAG and fixation type was observed.

**Conclusions:**

Although fixation type influenced absolute age predictions, it did not significantly modify RAG–disease associations. These findings support the use of either fixation type in oculomics research where imaging flexibility is required, although formal interchangeability cannot be claimed given the systematic bias, heteroscedasticity, and subgroup variation in agreement identified. Prospective validation in population-based cohorts is required before clinical application can be achieved.

The retina offers a unique, noninvasive window into an individual's systemic health, reflecting both ocular and physiological aging processes.^1^^–^^7^ Chronological age, determined by birth date, often diverges from biological age, which captures the body's physiological state and its associated age-related risk of adverse outcomes.^8^^–^^12^

Deep learning (DL) models applied to color fundus photographs (CFPs) can predict an individual's chronological age.^1^^,^^3^ The difference between the model-predicted age and an individual's observed chronological age can be interpreted in two distinct ways. In the context of model evaluation, this difference is considered a prediction error, quantified using mean absolute error (MAE), and reflects reduced model accuracy. In oculomics, where systemic conditions are associated with measurable retinal or ocular changes,^7^ the same difference is reframed as the retinal age gap (RAG), defined as predicted age minus chronological age. Rather than a model failure, RAG is hypothesized to reflect a biologically meaningful signal, with larger positive values suggesting accelerated retinal aging. This distinction between prediction error and biological signal is particularly important when a model trained on healthy individuals is applied to a clinical cohort. RAG has been associated with increased risks of mortality, cardiovascular and metabolic diseases, stroke, kidney failure, Parkinson's disease, schizophrenia, and ophthalmic conditions such as diabetic retinopathy (DR).^1^^,^^3^^,^^13^^–^^18^ Both macula- and disc-centered CFPs are widely used in clinical practice and research. For example, DR screening in England uses both disc- and macula-centered images, and disc-centered images are commonly used in prospective trials and fundus image datasets globally.^19^^–^^22^ The choice between these imaging approaches often depends on clinical protocols.

Despite the widespread use of both imaging types, no studies have directly compared the performance and agreement between macula- and disc-centered CFPs in predicting age. Macula-centered CFPs focus on the macula, which contains the highest density of photoreceptors and is particularly vulnerable to metabolic stress.^23^ Disc-centered CFPs capture the optic nerve head and major retinal vessels, where early vascular changes and neurodegeneration are often first apparent.^24^^,^^25^ We hypothesize that anatomic and physiological differences between retinal areas captured by macula- and disc-centered CFPs may contribute differently to age-related changes detectable by DL algorithms; therefore, they may influence their performance in age prediction and RAG-based disease associations.

Understanding these differences is important for optimizing oculomic applications and guiding clinical imaging protocols. This study addresses this gap by comparing DL-based age prediction using macula- and disc-centered CFPs in a large and diverse retrospective cohort. Our objective was to compare the prediction performance, inter-method agreement, and RAG-disease associations of both macula- and disc-centered CFPs. As a secondary objective, we assessed whether the relationship of RAG with ocular and systemic diseases was modified by image fixation (the retinal location on which the camera is centered during image acquisition).

## Methods

### Cohort and Participants

This retrospective cohort study was conducted within the AlzEye cohort, which includes 353,157 individuals aged 40 years and older who visited Moorfields Eye Hospital NHS Foundation Trust in London, United Kingdom, between January 1, 2008, and March 31, 2018. The AlzEye dataset integrates longitudinal retinal imaging data with systemic health information from the Hospital Episode Statistics (HES) Admitted Patient Care Database, coded using the International Classification of Diseases, 10th revision (ICD-10). The AlzEye study has received institutional and ethical review board approval (REC reference: 18/LO/1163). Detailed descriptions of the study design, governance, and cohort characteristics have been previously reported.^26^

### Fundus Imaging

For this analysis, a subset of participants with same-day macula- and disc-centered CFPs was selected. All images were acquired with a 45-degree field of view using the Topcon 3D OCT-2000SA system. All patients with at least one same-day macula- and disc-centered CFP pair were included. No restriction was applied to the number of image pairs per patient. Where patients contributed multiple pairs across visits, all eligible pairs were retained. Images underwent quality assessment and poor-quality images were excluded (details provided below in the Retinal Age Gap section).

### Retinal Age Gap

To calculate RAG, chronological age was first predicted from CFPs in our cohort. We used a previously described DL model that uses CFPs to predict chronological age.^3^^,^^27^ The model was trained on 133,894 fundus images, including both macula- and disc-centered images, from 29,003 individuals aged 15 to 91 years, with no reported ophthalmic or systemic diseases. These participants were drawn from four cohorts, including one from the UK Biobank and three cohorts from China. Importantly, the training dataset comprised both macula- (*n* = 117,959, 88.1%) and disc-centered images (*n* = 15,935, 11.9%).

The model incorporates an automated, previously described quality assessment component^28^ that evaluates image quality, and images of poor quality were excluded from analysis prior to age prediction. Age prediction was performed independently for macula- and disc-centered images. The RAG was calculated as the age predicted by the DL model minus the participant's chronological age at the time of fundus imaging. A greater RAG therefore indicates a predicted age higher than the chronological age.

### Clinical Variables

Patient characteristics and clinical conditions were extracted from the AlzEye dataset to stratify the cohort for subgroup analyses and to adjust for potential confounders in evaluating the performance of DL-based age predictions from macula- and disc-centered CFPs. Demographic variables included chronological age, sex, and ethnicity, categorized according to the UK Census as Asian/Asian British, White, Black/Black British, Mixed, Other, Unknown, or Missing. Clinical conditions included ophthalmic and systemic diagnoses. Ophthalmic conditions included glaucoma, age-related macular degeneration (AMD), nonproliferative diabetic retinopathy (NPDR), and proliferative diabetic retinopathy (PDR) as defined previously.^26^ Systemic conditions included diabetes mellitus (without retinopathy), dementia, hypertension, and major adverse cardiovascular events (MACEs). These conditions were identified using hospital admission records from the HES Admitted Patient Care Database, coded with the ICD-10.^26^

### Statistical Analysis

The reporting of inter-method reliability and agreement in this study follows the Guidelines for Reporting Reliability and Agreement Studies (GRRAS).^29^ Statistical analyses were conducted using Python software (version 3.11.3) with the SciPy and NumPy libraries for computational tasks,^30^^,^^31^ and R software (version 4.2.3) with the blme package for all mixed-effects modeling.^32^^,^^33^

To evaluate chronological age prediction performance from macula- and disc-centered CFPs, the MAE was calculated, with 95% confidence intervals (CIs) estimated via bootstrap resampling (1000 iterations and percentile method).^34^ Paired *t*-tests compared DL-predicted ages from macula- and disc-centered images.^35^ Agreement between predictions from macula- and disc-centered images was assessed using intraclass correlation coefficients (ICC) derived from a two-way mixed-effects model.^36^ Bland–Altman analysis was performed to determine bias and 95% limits of agreement (LoA; mean difference ± 1.96 × SD of differences). Bias was defined as the mean difference between age predictions derived from macula-centered CFPs and those from disc-centered CFPs (macula-centered predictions minus disc-centered predictions). To ensure the range of values in our study was sufficiently wide and avoid spuriously good agreement in the Bland–Altman analysis, we applied the Preiss-Fisher procedure as recommended for method comparison studies.^37^ This was performed using an online calculator based on the updated Preiss-Fisher method.^38^

To investigate associations between RAG and the presence or absence of ophthalmic and systemic health conditions (as defined earlier), generalized linear mixed-effects models (GLMMs) with a binomial error distribution and logit link function were fitted using the blme package.^32^^,^^33^ The models were fitted on a long-format dataset in which each row represents a single RAG value from a single image, giving 24,460 observations in total across 5895 patients. Each model included RAG (standardized to mean = 0, SD = 1), fixation type, their interaction, chronological age (centered at the sample mean), and sex as fixed effects, with a random intercept for participant ID (1 | id) to account for within-person correlation. The primary exposure of interest was the RAG: fixation interaction. Because RAG was strongly negatively correlated with chronological age (Pearson's *r* = −0.77) and several outcomes had low prevalence (<5%), we used a stabilized estimation method using the bglmer function in the blme package with informative priors designed to improve numerical stability and reduce extreme estimates.^32^^,^^33^ It should be noted that for conditions with low event rates, including dementia (1.2%), diabetes without retinopathy (2.8%), and PDR (3.2%), statistical power to detect an RAG: fixation interaction may have been limited. Separate models were fitted for each condition. Odds ratios (ORs) with 95% Wald CIs and 2-sided *P* values were reported. Statistical significance was set at 2-sided *P* < 0.05. As a sensitivity analysis, all GLMMs were additionally refitted after restricting the dataset to one image pair per patient (first available visit, *n =* 5895), using the same model specification.

Predefined subgroup analyses stratified by sex, age, ethnicity, and clinical conditions evaluated variations in MAE, ICC, and Bland–Altman metrics. Missing ethnicity data were treated as a distinct category in subgroup analyses.

### Data Availability

National and international collaborations are welcomed; however, the data are subject to the contractual restrictions of the data sharing agreements among National Health Service Digital, Moorfields Eye Hospital, and University College London and are therefore not available for access beyond the AlzEye research team. Researchers should contact the Chief Investigator at p.keane@ucl.ac.uk.

## Results

### Cohort Description

From an initial AlzEye cohort of 353,157 patients, 154,830 (43.8%) underwent retinal imaging. Of these, 6571 patients (4.2%) had same-day macula- and disc-centered CFPs and were eligible for analysis. These 6571 patients had 14,083 same-day image pairs, with some patients contributing multiple image pairs throughout the study period. After excluding image pairs in which either image failed the model's automated quality assessment,^28^ the final dataset comprised 5895 unique patients with 12,230 same-day macula- and disc-centered image pairs (24,460 individual images).

At the image-pair level, the cohort had a balanced sex distribution and spanned a wide age range, with nearly equal representation across age groups (Table 1). The ethnic diversity reflects the multicultural population served by Moorfields Eye Hospital, although a substantial proportion of image pairs (20.2%) had missing ethnicity data. Hypertension was the most prevalent systemic condition (38.5%), whereas glaucoma and NPDR were the most common ophthalmic conditions (17.1% and 15.1%, respectively). Image pairs from patients with diabetes accounted for 21.1% of the cohort and were stratified by retinopathy status into NPDR (15.1%), PDR (3.2%), and diabetes without retinopathy (2.8%).

**Table 1.** Baseline Characteristics of Image Pairs

Category	Subgroup	*n* (%)	Mean Age (SD)
All		12,230 (100.0%)	61.9 (13.1)
Sex	F	6,403 (52.4%)	62.1 (13.5)
	M	5,827 (47.6%)	61.7 (12.6)
Age, y	≤50	2,508 (20.5%)	44.1 (3.8)
	50–60	3,153 (25.8%)	55.2 (2.8)
	60–70	3,073 (25.1%)	64.7 (2.9)
	≥70	3,496 (28.6%)	78.1 (5.7)
Ethnicity	Asian/Asian British	2,096 (17.1%)	63.4 (12.3)
	White	4,238 (34.6%)	63.9 (14.0)
	Black/Black British	955 (7.8%)	62.2 (12.3)
	Mixed	105 (0.9%)	62.9 (12.9)
	Other	1,842 (15.1%)	59.6 (11.9)
	Unknown	518 (4.2%)	60.1 (11.6)
	Missing	2,476 (20.2%)	59.1 (12.8)
Ophthalmic conditions	Glaucoma	2,091 (17.1%)	65.9 (11.7)
	AMD	268 (2.2%)	75.0 (10.8)
	NPDR	1,849 (15.1%)	62.0 (11.9)
	PDR	397 (3.2%)	56.5 (9.2)
Systemic conditions	Diabetes without retinopathy	346 (2.8%)	64.1 (12.3)
	Dementia	149 (1.2%)	79.0 (8.6)
	Hypertension	4,709 (38.5%)	68.7 (12.0)
	MACE	948 (7.8%)	70.5 (12.7)

AMD, age-related macular degeneration; MACE, major adverse cardiovascular events; *n*, number of same-day image pairs; NPDR, nonproliferative diabetic retinopathy; PDR, proliferative diabetic retinopathy; SD, standard deviation.

### Chronological Age Prediction Performance

When predicting chronological age in this cohort, macula-centered predictions had a smaller MAE than disc-centered predictions, with an MAE of 7.09 years (95% CI = 6.99–7.19) versus 7.77 years (95% CI = 7.66–7.88), respectively (Table 2). The paired comparison of age predictions between fixation types was statistically significant (*P* < 0.01). The lower MAE for macula-centered images was observed across most subgroups (see Table 2).

**Table 2.** Subgroup Analysis of Age Prediction Performance

Category	Subgroup	MAE Macula [95% CI]	MAE Disc [95% CI]	*P* Value
All		7.09 [6.99, 7.19]	7.77 [7.66, 7.88]	<0.01
Sex	F	7.32 [7.18, 7.47]	7.91 [7.76, 8.07]	<0.01
	M	6.83 [6.70, 6.98]	7.60 [7.45, 7.77]	<0.01
Age, y	≤50	10.60 [10.34, 10.88]	12.45 [12.18, 12.71]	<0.01
	50–60	6.59 [6.43, 6.75]	7.80 [7.63, 7.98]	<0.01
	60–70	4.05 [3.93, 4.17]	4.47 [4.34, 4.59]	<0.01
	≥70	7.69 [7.50, 7.87]	7.27 [7.09, 7.45]	<0.01
Ethnicity	Asian/Asian British	6.00 [5.79, 6.21]	6.44 [6.22, 6.65]	<0.01
	White	7.71 [7.55, 7.89]	8.42 [8.23, 8.60]	<0.01
	Black/Black British	6.45 [6.12, 6.79]	6.96 [6.60, 7.31]	<0.01
	Mixed	5.95 [5.07, 6.83]	6.95 [5.98, 7.97]	0.91
	Other	6.70 [6.46, 6.93]	7.66 [7.41, 7.91]	<0.01
	Unknown	6.25 [5.83, 6.69]	6.86 [6.46, 7.31]	<0.01
	Missing	7.71 [7.47, 7.97]	8.40 [8.15, 8.67]	<0.01
Ophthalmic Conditions	Glaucoma	6.45 [6.21, 6.67]	6.83 [6.59, 7.07]	<0.01
	AMD	9.35 [8.59, 10.14]	10.03 [9.14, 10.95]	0.462
	NPDR	7.31 [7.07, 7.56]	7.92 [7.64, 8.18]	<0.01
	PDR	9.71 [9.14, 10.28]	10.41 [9.77, 11.08]	<0.01
Systemic Conditions	Diabetes without retinopathy	6.72 [6.20, 7.23]	6.71 [6.18, 7.29]	<0.01
	Dementia	8.88 [7.90, 9.96]	8.76 [7.89, 9.78]	0.199
	Hypertension	7.12 [6.96, 7.27]	7.27 [7.11, 7.43]	<0.01
	MACE	7.91 [7.56, 8.26]	7.80 [7.46, 8.15]	<0.01

CI, confidence interval; MAE, mean absolute error.

*P* values compare DL-predicted ages between macula- and disc-centered images.

Age-stratified analyses revealed the highest MAE occurred in individuals aged ≤50 years (macula = 10.60; disc = 12.45), and the lowest MAE in those aged 60 to 70 years (macula = 4.05; disc = 4.47). Among clinical subgroups, patients with PDR exhibited the highest MAE (macula = 9.71; disc = 10.41). Notably, age predictions did not significantly differ between imaging types in patients with AMD, dementia, or those of mixed ethnicity.

### Agreement Between Prediction Methods

Overall agreement between macula- and disc-centered predictions was high, with an ICC of 0.83 (95% CI = 0.82–0.84; Table 3). The weakest ICCs were observed in individuals aged 50 to 60 years, 60 to 70 years, and in patients with dementia and PDR. The strongest agreement was seen in Asian/Asian British, Black/Black British, and mixed ethnicity groups.

**Table 3.** Agreement Analysis Between Macula-Centered and Disc-Centered Age Predictions

Category	Subgroup	ICC [95% CI]
All		0.83 [0.82, 0.84]
Sex	F	0.83 [0.82, 0.84]
	M	0.83 [0.82, 0.84]
Age, y	≤50	0.70 [0.67, 0.72]
	50–60	0.67 [0.65, 0.69]
	60–70	0.59 [0.55, 0.63]
	≥70	0.70 [0.68, 0.72]
Ethnicity	Asian/Asian British	0.87 [0.85, 0.88]
	White	0.83 [0.82, 0.84]
	Black/Black British	0.86 [0.84, 0.87]
	Mixed	0.88 [0.82, 0.92]
	Other	0.79 [0.75, 0.82]
	Unknown	0.84 [0.81, 0.87]
	Missing	0.80 [0.78, 0.82]
Ophthalmic conditions	Glaucoma	0.82 [0.81, 0.84]
	AMD	0.81 [0.75, 0.85]
	NPDR	0.81 [0.79, 0.83]
	PDR	0.71 [0.64, 0.76]
Systemic conditions	Diabetes without retinopathy	0.82 [0.77, 0.85]
	Dementia	0.73 [0.64, 0.80]
	Hypertension	0.83 [0.82, 0.84]
	MACE	0.80 [0.77, 0.83]

ICC, intraclass correlation coefficient.

**Table 4.** Mixed-Effects Model Results

Category	Condition	OR RAG (95% CI)	*P* Value	OR Fixation Type (95% CI)	*P* Value	OR RAG: Fixation Type (95% CI)	*P* Value
Ophthalmic conditions	Glaucoma	1.32 (0.69, 2.52)	0.404	0.96 (0.56, 1.65)	0.894	0.99 (0.58, 1.71)	0.982
	AMD	3.57 (1.76, 7.24)	**<0.001**	0.88 (0.49, 1.58)	0.664	0.96 (0.58, 1.60)	0.885
	NPDR	1.46 (0.70, 3.06)	0.313	0.95 (0.52, 1.77)	0.882	0.97 (0.52, 1.83)	0.928
	PDR	2.30 (0.31, 16.9)	0.414	0.85 (0.11, 6.79)	0.879	1.01 (0.18, 5.86)	0.988
Systemic conditions	Diabetes without retinopathy	1.13 (0.22, 5.85)	0.883	0.95 (0.24, 3.73)	0.938	0.99 (0.24, 4.08)	0.990
	Dementia	0.99 (0.11, 8.76)	0.991	0.86 (0.07, 10.50)	0.908	0.92 (0.14, 6.05)	0.934
	Hypertension	1.37 (0.78, 2.41)	0.268	0.96 (0.69, 1.33)	0.800	0.98 (0.63, 1.52)	0.915
	MACE	1.23 (0.44, 3.43)	0.689	0.96 (0.39, 2.39)	0.931	0.98 (0.43, 2.21)	0.953

OR, odds ratio; RAG, retinal age gap.

Values in bold indicate statistical significance. ORs represent the change in odds per 1-SD increase in RAG, the difference in odds for disc versus macula fixation (fixation type), and the RAG: fixation type interaction.

### Visualization of Agreement

The Figure presents Bland–Altman plots assessing agreement between macula- and disc-centered predictions. The mean bias was −1.13 (SD = 4.69), with LoA ranging from −10.32 to 8.07. The plots showed heteroscedasticity, with widening differences around middle-aged predictions and narrower differences at younger and older extremes.

**Figure.**
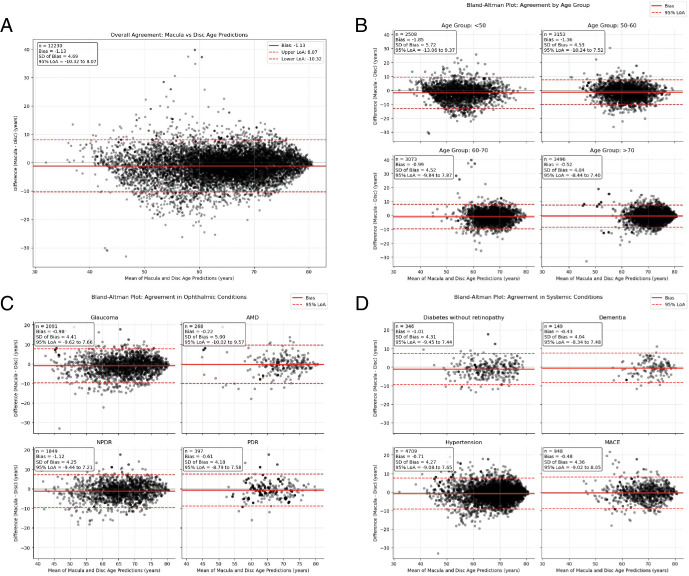
Bland–Altman plots demonstrating agreement between macula- and disc-centered retinal age predictions. (**A**) Whole study population. (**B**) Results stratified by age groups. (**C**) Stratification by ophthalmic conditions. (**D**) Stratification by systemic conditions. In all panels, each point represents the difference between predictions plotted against their average. The *solid horizontal lines* represent the mean bias, whereas the *dashed lines* indicate the 95% limits of agreement.

Across ophthalmic and systemic subgroups, similar heteroscedastic patterns and negative biases were observed in most subgroups, indicating that macula-centered predictions were consistently lower than disc-centered predictions.

### Preiss-Fisher Analysis

Preiss-Fisher analysis indicated that the range of measurements in this cohort was sufficient for Bland–Altman analysis. The original *R*² between the paired values was 0.95. Across 100 random mispairing iterations, the average *R*² was 0.84*.* Because the original *R*² fell outside the mean ± 2 SDs of the mispaired *R*² distribution, the observed agreement was unlikely to be spurious due to a narrow data range*.*

### Mixed-Effects Model Results: Associations Between Retinal Age Gap and Conditions

A larger RAG was significantly associated with increased odds of AMD (*OR* = 3.57, 95% CI = 1.76–7.24, *P* < 0.001; Table 4). For the remaining conditions, there was no evidence of association with RAG. Although several estimates were above 1, CIs were wide and all crossed unity, indicating substantial uncertainty.

For fixation type and the RAG, fixation interactions were nonsignificant for all conditions examined (all *P* > 0.05), indicating that the association between RAG and ophthalmic or systemic diseases was not modified by fixation.

## Discussion

Despite the growing use of retinal age prediction algorithms in clinical research and their emerging deployment in health screening,^39^^,^^40^ it remains unclear whether image fixation affects performance. In this analysis of 12,230 same-day macula- and disc-centered CFP pairs acquired from an ethnically and socioeconomically diverse cohort of 5895 unique patients, we report the following key findings: first, lower prediction error (i.e. better performance) was observed in macula-centered images compared with disc-centered images. Second, overall inter-method agreement was high (ICC = 0.83), although Bland–Altman analysis revealed systematic negative bias and heteroscedasticity. Third, the relationship between RAG and both ophthalmic and systemic diseases was not significantly modified by fixation type. Our results suggest that macula- and disc-centered CFPs yield broadly comparable results in oculomics research, supporting the use of either fixation type where flexibility is required. However, the systematic bias, heteroscedasticity, and subgroup variation in agreement identified here preclude a formal claim of interchangeability. Equally, for the nonsignificant RAG, the fixation interaction should not be interpreted as positive evidence of equivalence.

MAE was higher for both macula- and disc-centered CFPs than in the original model-development study, which reported an MAE of 2.79 years.^27^ This performance gap may reflect differences in cohort characteristics. The training data primarily drew from population-based cohorts such as the UK Biobank, which consist of generally healthier individuals with less comorbidity compared to our hospital-based AlzEye cohort.^26^ Additionally, the training dataset’s imbalance toward macula-centered images (88.1% vs. 11.9% disc-centered) may contribute to the superior performance of macula-centered predictions and could explain the observed MAE differences between modalities. Furthermore, differences in imaging equipment (our exclusive use of Topcon systems versus varied devices in training), hospital center, ethnic diversity, and age distributions may also contribute to reduced generalizability, leading to higher external validation errors. The substantially higher MAE observed in this hospital-based cohort suggests that the model performs less accurately when applied to eyes with disease-related structural changes not seen during training, raising the possibility that elevated RAG in this cohort partly reflects this inaccuracy rather than accelerated biological aging exclusively.

Agreement between age predictions from macula- and disc-centered images was generally high (ICC = 0.83, 95% CI = 0.82–0.84), indicating good reliability across fixation types, despite anatomic differences. Heteroscedasticity was evident, with greater disagreement in the middle age range, suggesting that the two methods are not uniformly equivalent across the age spectrum. Agreement was reduced among patients aged 50 to 60 years (ICC = 0.67) and 60 to 70 years (ICC = 0.59), and among those with proliferative DR (ICC = 0.71) or dementia (ICC = 0.73). Both fixation types are therefore suitable for population-level research, but fixation choice requires more careful consideration in studies focused on middle-aged patients or those with advanced retinal disease.

Larger positive RAG was associated with higher odds of AMD. This association is compatible with the hypothesis that RAG captures aspects of accelerated retinal aging. However, as discussed above, in a hospital-based cohort with prevalent structural pathology, elevated RAG may also partly reflect prediction error in eyes with structural pathology not represented in the training data, rather than biological aging exclusively. For the RAG, fixation interaction was nonsignificant across all conditions examined. However, due to low prevalence of some conditions, nonsignificant interactions may reflect insufficient power. Nevertheless, this supports the use of either macula- or disc-centered imaging for RAG-based research where imaging flexibility is required.

The strong negative correlation between RAG and chronological age (*r* = −0.77) observed in this cohort indicates systematic age-dependent prediction bias. The model tended to overestimate age in younger individuals and underestimate it in older ones. Inclusion of chronological age as a fixed-effect covariate in the GLMMs provides a correction for this bias. However, a linear adjustment may not fully account for a nonlinear calibration error across the age spectrum. The age-stratified MAE data support this, with the highest errors observed in the youngest (≤50 years: MAE 10.60 macula, 12.45 disc) and oldest (≥70 years: MAE 7.69 macula, 7.27 disc) age groups.

The GLMM random intercept for participant ID accounts for within-person clustering but does not fully capture the hierarchical nature of the data, in which some patients contributed observations from both eyes and multiple visits. As a sensitivity analysis, models were refitted after restricting to one image pair per patient (first available visit, *n* = 5,895; Supplementary Table S1). Results were broadly consistent with the primary analysis, with the RAG fixation interaction remaining nonsignificant for all stable models. However, the RAG OR for AMD was no longer statistically significant in the sensitivity analysis, likely reflecting attenuation due to removal of patients with multiple visits who are enriched for established AMD. We explored more complex random-effects structures, including an eye-within-visit-within-patient random intercept and a three-level structure incorporating eye laterality: (1 | id) + (1 | visit) + (1 | eye). However, these models yielded unstable results, likely due to insufficient data at these nested levels to reliably estimate additional random effects. The primary two-level model therefore represents the most stable and feasible specification for this dataset. Future work with larger samples retaining multiple visits per patient may be better placed to implement these structures reliably.

All imaging was acquired at a single tertiary ophthalmology center using one camera system, which may limit generalizability to other clinical settings. The hospital-based nature of the cohort also means that disease prevalence, severity, and image quality differ substantially from population-based screening settings, and prospective validation in such settings is necessary before these findings can inform clinical imaging protocols. A strength is the ethnic diversity of the AlzEye cohort, which includes substantial representation of Asian, Black, and other minority groups, and may improve applicability to similarly diverse urban populations.

## Conclusions

This study suggests that although DL-based age prediction using macula-centered images yields lower prediction error, macula- and disc-centered fixation types provide broadly comparable inter-method agreement, and no statistically significant modification of RAG–disease associations by fixation type was observed in this cohort. However, the systematic bias, heteroscedasticity, and subgroup variation in agreement identified here preclude a formal claim of interchangeability, and the absence of a significant RAG: fixation interaction should not be interpreted as positive evidence of equivalence given the limitations of this study, particularly for low-prevalence conditions. These findings support the use of either fixation type in oculomics research where imaging flexibility is required. However, prospective validation in population-based and clinically diverse cohorts is necessary before conclusions can be drawn about the biological significance of RAG or its utility in clinical health monitoring.

## Supplementary Material

Supplement 1
