# Supplementary material for: Macula- Versus Disc-Centered Fundus Photography: Performance in Age-Prediction and Disease Associations
Source: Invest Ophthalmol Vis Sci. 2026 Jun 15;67(6):24. doi: 10.1167/iovs.67.6.24 (PMC13277792; doi:10.1167/iovs.67.6.24)
Supplement: Supplement 1 [file iovs-67-6-24_s001.docx]

Supplementary Table S1. Sensitivity analysis of mixed-effects model results restricted to the first image pair per patient (n = 5,895 patients; 11,790 observations).

| **Category** | **Condition** | **OR RAG (95% CI)** | ***p*-value** | **OR Fixation Type (95% CI)** | ***p*-value** | **OR RAG : Fixation Type (95% CI)** | ***p*-value** |
| --- | --- | --- | --- | --- | --- | --- | --- |
| **Ophthalmic conditions** | Glaucoma | Unstable† | — | Unstable† | — | Unstable† | — |
|  | AMD | 0.73 (0.13–4.00) | 0.717 | 0.93 (0.15–5.65) | 0.921 | 0.93 (0.24–3.67) | 0.919 |
|  | NPDR | 1.42 (0.71–2.85) | 0.325 | 0.96 (0.53–1.73) | 0.882 | 0.97 (0.53–1.75) | 0.913 |
|  | PDR | 2.28 (0.35–15.00) | 0.392 | 0.87 (0.11–6.93) | 0.868 | 0.99 (0.19–5.26) | 0.993 |
| **Systemic conditions** | Diabetes without retinopathy | 1.07 (0.21–5.37) | 0.934 | 0.96 (0.24–3.82) | 0.938 | 1.00 (0.25–4.02) | 0.996 |
|  | Dementia | Unstable† | — | Unstable† | — | Unstable† | — |
|  | Hypertension | 1.56 (0.86–2.82) | 0.146 | 0.94 (0.70–1.27) | 0.641 | 0.97 (0.64–1.46) | 0.869 |
|  | MACE | 1.17 (0.43–3.17) | 0.753 | 0.96 (0.41–2.28) | 0.931 | 0.97 (0.45–2.13) | 0.947 |

Model specification: outcome ~ RAG_scaled × fixation + age_centered + sex + (1 | id). Models were fitted as two-level mixed-effects logistic regression models using Bayesian regularisation via bglmer.

Fixation type OR represents disc-centered versus macula-centered color fundus photographs. ORs for RAG represent the change in odds per 1-SD increase in RAG.

† Glaucoma and dementia sensitivity models produced near-zero standard errors across fixed effects (SE ≈ 0.0007), consistent with unstable model output likely related to separation/sparse outcomes. These rows are therefore marked as unstable and should not be interpreted as reliable effect estimates.

For all stable models, the RAG : fixation type interaction was not statistically significant (all P > 0.05). The AMD RAG OR was lower and non-significant in this first-pair sensitivity analysis than in the primary analysis, likely reflecting attenuation after removal of repeat visits from patients enriched for established AMD.
